# Level IV neck dissection in cN0 HPV-negative oropharyngeal squamous cell carcinoma: a retrospective cohort study

**DOI:** 10.1186/s12885-022-09609-x

**Published:** 2022-05-12

**Authors:** Zirong Huo, Shuiting Fu, Chunyue Ma, Surui Sheng

**Affiliations:** 1 Department of Otorhinolaryngology Head and Neck Surgery, Shanghai Ninth People’s Hospital, Shanghai Jiaotong University School of Medicine, Shanghai, 200011 China; 2 Department of Oral & Maxillofacial – Head & Neck Oncology, Shanghai Ninth People’s Hospital, College of Stomatology, Shanghai Jiao Tong University School of Medicine; National Center for Stomatology; National Clinical Research Center for Oral Diseases; Shanghai Key Laboratory of Stomatology, 639, Zhi Zao Ju Road, Shanghai, 200011 China

**Keywords:** Oropharyngeal squamous cell carcinoma (OPSCC), Human papillomavirus (HPV), Selective neck dissection (SND), Disease-free survival (DFS), Overall survival (OS), Disease-specific survival (DSS)

## Abstract

**Background:**

As opposed to observation of the neck, elective neck dissection has a survival benefit for cN0 oropharyngeal squamous cell carcinoma (OPSCC). However, there are limited date on level IV neck dissection in human papillomavirus (HPV)-negative OPSCC because most earlier studies did not stratify by P16 or HPV status. Thus, whether to exclude level IV from selective dissection (SND) of cN0 HPV-negative OPSCC remains controversial.

**Methods:**

In this single-center retrospective cohort study, disease-free survival (DFS) was estimated as the primary endpoint for 124 cN0 HPV-negative OPSCC patients who received SND of levels I-III (Group A) and I-IV (Group B). Overall survival (OS) and disease-specific survival (DSS) were considered secondary endpoints.

**Results:**

For the entire cohort, the 5-year DFS rates of Groups A and B were 55.0% and 60.1%, respectively. Five-year OS rates were 58.9% and 61.5%, and 5-year DSS rates were 74.0% and 64.8%, respectively. Group B did not show higher 5-year DFS, OS, or DSS than Group A.

**Conclusions:**

This retrospective cohort study validated that in cN0 HPV-negative OPSCC, SND including level IV does not have substantial benefits regarding DFS, OS or DSS.

## Background

Oropharyngeal squamous cell carcinoma (OPSCC), mostly comprising poorly differentiated primary carcinoma, has a marked propensity for lymphatic spread, even in early stages. Rates of occult lymph node metastasis in cN0 OPSCCs can exceed 20% [1, 2]. Compared surgical resection of the primary tumor with observation of the neck, patients of cT1-cT3 cN0 OPSCC performed with elective neck dissection (END) have higher 5-year overall survival (OS), disease-free survival (DFS) and disease-specific survival (DSS) rates [3].

OPSCC frequently metastasizes to levels II, III and IV. Thus, selective neck dissection (SND) of II-IV is suggested for OPSCC of cN0 neck in traditional guidelines [4–6]. Nevertheless, controversy remains about dissecting level IV cN0 OPSCC. Supraomohyoid neck dissection (level I-III) is also recommended because pathological lymph nodes in OPSCC are more frequent at level I than level IV [7, 8].

In addition, data on level IV neck dissection with regard to human papillomavirus (HPV)-negative OPSCC are limited. Most early studies did not stratify patients by p16 or HPV status. Recently, focus has been on HPV-positive OPSCC due to its increasing incidence in Western countries; the majority of cases in the United States are potentially HPV positive, particularly among recent birth cohorts, perhaps resulting from changes in sexual behaviors. [9–12]. The majority of OPSCCs in some regions, such as China, are still HPV negative [13, 14]. Although the distribution of lymph node drainage between HPV-positive and HPV-negative OPSCC is not meaningful, there is a higher prevalence of pN + OPSCC in the HPV-positive group [15–17].

Importantly, OPSCC patients with lymph node metastasis or recurrence at level IV have significantly worse 5-year DFS than patients with metastasis to other neck levels (54% vs. 71%) [2]. Therefore, whether to exclude level IV from routine neck dissection in cN0 HPV-negative OPSCC is a potential issue, and an appropriate neck dissection paradigm needs to be defined. Evidence to date mainly originates from observational studies, whereas large-sample retrospective or prospective cohort studies on level IV neck dissection associated with survival outcomes in HPV-negative OPSCC are lacking. This study retrospectively evaluated the appropriate SND of level IV with cN0 HPV-negative OPSCC by comparing DFS, OS and DSS in a single-center cohort.

## Methods

A neck stage of cN0 was defined through palpation, ultrasound, and CT/MRI scans before surgery. All patients had received examination of ultrasound and CT/MRI scans (each patients received either CT or MRI scans or both) before surgery. PET-CT was not used for all OPSCC patients for lymph node evaluation. Stage was diagnosed as cN + by ultrasound when one or more of the following were present: absent hilus, inhomogeneous echo structure, volume > 9 mm^3^, longitudinal diameter > 18 mm, maximal transverse diameter > 8 mm and ratio of transverse to longitudinal diameter > 0.67 [18]. For CT or MRI criteria, lymph nodes manifesting central nodal necrosis larger than 10 mm in the short axis, irregular nodal boundaries and obliteration of the adjacent fat planes were considered cN + .

### Study population and definition

**Fig. 1:**
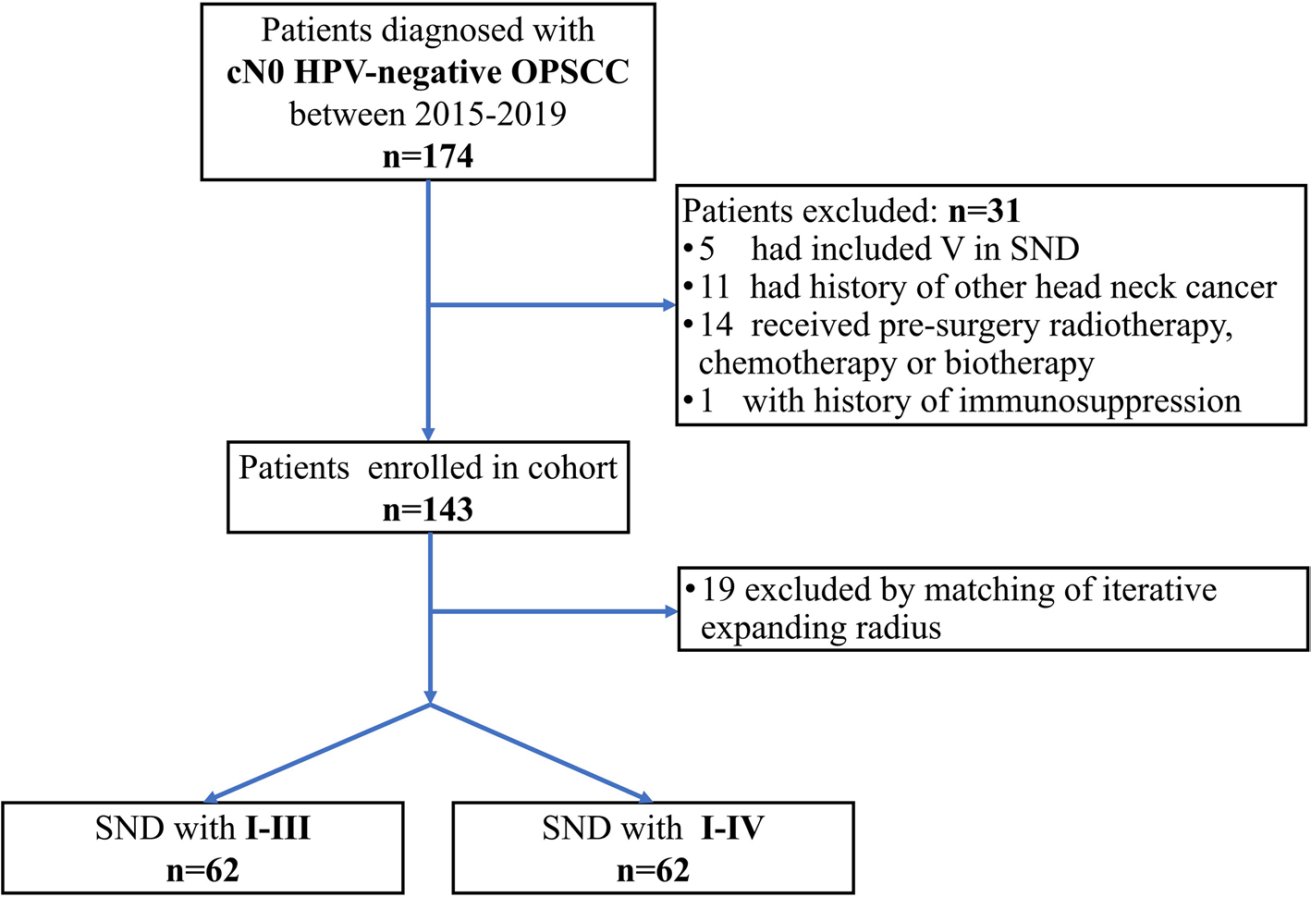
Diagram illustrates derivation of study cohort

The single-center retrospective cohort study enrolled 124 consecutive patients with HPV-negative OPSCC from Ninth People's Hospital affiliated to Shanghai Jiao Tong University School of Medicine from 1 January 2015 to 31 January 2019 (Fig. 1). OPSCC was defined as primary located in base of tongue, tonsil, soft palate or lateral/posterior pharyngeal walls. OPSCC, which was arisen from oral cavity and then invaded oropharynx such as tongue and hard palate, were not considered as OPSCC. All OPSCC patients underwent surgery with primary resection (not transoral robotic surgery) and unilateral or bilateral SND. Eighty-three patients (66.9%) were reconstructed with free flap and 20 patients (16.1%) were reconstructed with adjacent flap. One hundred and two patients (82.3%) received tracheotomy. OPSCC was diagnosed by pathology, and HPV status was determined by immunochemistry (IHC) staining of p16 and HPV-DNA in situ hybridization (ISH). According to the College of American Pathologists Guideline, when nuclear and cytoplasmic expression of p16 was more than 70% by IHC, the tumor was diagnosed as HPV-positive [19]. In our study, when expression of p16 was negative, ISH was not conducted and the tumor was diagnosed as HPV-negative. If the expression of p16 was positive, even less than 70%, ISH was also conducted for HPV genotyping. And the HPV genotype of tumor would be categorized by ISH regardless of the percentage of p16 expression. Cases were categorized according to the 8th Edition of the UICC-AJCC TNM staging system. Recurrence of lymph nodes was confirmed by fine-needle aspiration biopsy or histopathological examination from second neck dissection. Radiotherapy was administered to patients who had one or more risk factors, including pT3/pT4 stage, positive margins, pN2/pN3 stage, lymphovascular invasion (LVI), and perineural invasion (PNI). Chemoradiotherapy was implemented for OPSCC at stage IVA-IVB.

This study was performed in accordance with the Declaration of Helsinki and received ethical approval from the Ninth People's Hospital affiliated with Shanghai Jiao Tong University School of Medicine (SH9H-2020-T407-1).

### Cohort selection and outcome

All patients received ipsilateral SND with I-III or I-IV, which was determined at each surgeon’s discretion based on factors of tumor eradication and function. The patients were divided into two groups according to different ranges of SND: Group A (I-III) and Group B (I-IV). Group A was considered the control group (Table 1 & Fig. 1).

**Table 1:** Demographic information and cohort of HPV-negative OPSCC patients (*n* = 124)

Variable		In total	SND with I-III (*n* = 62)	SND with I-IV (*n* = 62)	P
		n (%)			
Age, yr		60.7 ± 12.0^a^	60.9 ± 11.3^a^	60.5 ± 11.7^a^	0.823^b^
Sex, female		17 (13.7)	6 (9.7)	11 (17.7)	0.296^c^
Subsite	Base of tongue	94 (75.9)	47 (75.9)	47 (75.9)	1.000^c^
	Soft palate	22 (17.7)	11 (17.7)	11 (17.7)	
	Tonsil	6 (4.8)	3 (4.8)	3 (4.8)	
	Pharyngeal walls	2 (1.6)	1 (1.6)	1 (1.6)	
T stage	T1	15 (12.1)	7 (11.3)	8 (12.9)	0.991^c^
	T2	36 (29.0)	18 (29.0)	18 (29.0)	
	T3	46 (37.1)	23 (37.1)	23 (37.1)	
	T4	27 (21.7)	14 (22.3)	13 (21.0)	
N stage	N0	76 (61.3)	36 (58.1)	40 (64.5)	0.750^c^
	N1	28 (22.6)	14 (22.6)	14 (22.6)	
	N2	16 (12.9)	10 (16.1)	6 (9.7)	
	N3	4 (3.2)	2 (3.2)	2 (3.2)	
Radiotherapy		61 (49.2)	32 (51.6)	29 (46.8)	0.590^c^
Chemoradiotherapy		20 (16.1)	11 (17.7)	9 (14.5)	0.625^c^

*Abbreviations*: *OPSCC* Oropharyngeal squamous cell carcinoma, *SND* Selective neck dissection, *P p* value

^a^Mean ± SD

^b^*t*-test

^c^Person’s chi-squared test

Group A was assembled from a pool of 69 patients and Group B from a pool of 74 patients. To avoid bias from direct comparisons between the two groups, patients in Group A were matched to Group B in a 1:1 ratio by iteratively expanding radius matching. Matching factors were in the order of priority, as follows: tumor subsite, pathological T stage and age. Progressive radius matching was used for T stage and age, and an ideal matching radius was attempted (identical T stage and age ± 3). If matched participants could not be found by the ideal radius for a given characteristic, the radius was iteratively increased to a predetermined maximum permissible radius (T stage ± 1 and age ± 6).

The primary endpoint was disease-free survival (DFS), which was defined as the time between surgery and the first local and distal recurrence or all-cause death. Data were censored at the end of last follow-up for patients who were still alive (February 11th, 2021).

Secondary endpoints included the following:

1. Overall survival (OS), as defined as the time between surgery and death due to any cause. Data were censored at the end of last follow-up for patients who were still alive (February 11th, 2021).
2. Disease-specific survival (DSS) was defined as the time between surgery and death caused by OPSCC. Data were censored at the end of last follow-up for patients who were still alive or the date of OPSCC-unrelated death (February 11th, 2021).

### Surgical complications

We recorded 4 kinds of surgical complications associated with neck dissection of level IV (Table 2). Two patients in the entire cohort (2/124, 1.6%) had complications of chylous leakage, all in Group B (2/124, 1.6%, *p* = 0.496) (Table 2). There was 1 patient in both Group A (1/64, 1.6%) and Group B (1/64, 1.6%, *p* = 1.000) who experienced phrenic nerve paralysis (Table 2). Compared with Group A (5/62, 8.1%), patients in Group B (7/62, 11.3%, *p* = 0.544) had a slightly higher incidence of hematoma (Table 2). Regarding infection, there were no differences between Group A (8/62, 12.9%) and Group B (8/62, 12.9%, *p* = 1.000) (Table 2).

**Table 2:** Morbidity of surgical complications after neck dissection in cohort

Variable	In total	SND with I-III (*n* = 62)	SND with I-IV (*n* = 62)	P
	n (%)			
Chylous leakage	2 (1.6)	0	2 (3.2)	0.496^a^
Phrenic nerve paralysis	2 (1.6)	1 (1.6)	1(1.6)	1.000^a^
Hematoma	12 (9.7)	5 (8.1)	7 (11.3)	0.544^b^
Wound infection	16 (12.9)	8 (12.9)	8 (12.9)	1.000^b^

*Abbreviations*: *SND* Selective neck dissection, *P p* value

^a^Fisher’s exact test

^b^Person’s chi-squared test

### Covariates and missing data

Information on patient demographics was obtained from electronic medical history, which included age, sex, tumor subsite, T stage, N stage, depth of invasion (DOI) pathologic grade, LVI, PNI, adjuvant radiotherapy and chemoradiotherapy. These data were promising risk factors for OPSCC and defined as control variables. Mean DOI was used for when such data were available. Missing HPV status and lymph node metastasis data were excluded.

### Statistical analysis

Experimental values for continuous variables were analyzed by t-test, and the results are expressed as the mean ± standard error and. Pearson’s chi-squared test and Fisher’s exact test were used to analyze categorical variables between groups. The Kaplan–Meier method with the log-rank test was applied to estimate OS, DFS and DSS in univariate analysis. Potential confounders of OS, DFS and DSS were adjusted by Cox proportional hazard models. A two-tailed P value of less than 0.05 was considered to indicate statistical significance. The statistical analysis was performed using SPSS 19.0.

## Results

One hundred and twenty-four HPV-negative OPSCC patients, including 17 females and 107 males (Table 1), were enrolled. Patient age ranged from 28 to 88 years old, and the average age was 60.7 ± 12.0 years (Table 1). Most OPSCCs were located in the base of the tongue (94, 75.9%) and soft palate (22, 17.7%) (Table 1). The distribution of OPSCCs for pathological T stage was T1 (15, 12.1%), T2 (36, 29.0%), T3 (46, 37.1%) and T4 (27, 21.7%) (Table 1). Regarding the histologic grade of tumors, 62 (50%) OPSCCs were categorized as well differentiated; 54 (43.5%) and 8 (6.5%) OPSCCs were moderately and poorly differentiated, respectively (Table 3). Fourteen (11.3%) OPSCCs had LVI, and 20 (16.1%) OPSCCs had PNI (Table 3). Sixty-one (49.2%) patients received adjuvant radiotherapy, and 20 (16.1%) patients received adjuvant chemoradiotherapy (Table 3). The choice of SND did not correlate with age (*p* = 0.823), sex (*p* = 0.296), tumor subsite (*p* = 1.000), T stage (*p* = 0.991), N stage (*p* = 0.750), radiotherapy (*p* = 0.590) or chemoradiotherapy (*p* = 0.625) (Table 1).

**Table 3:** Univariate analysis

Variables		n (%)	DFS		OS		DSS	
			**5-y** **(%)**	**P**	**5-y (%)**	**P**	**5-y** **(%)**	**P**
All		124	58.5		61.1		68.4	
Mean age, year	≤ 60	60.7 ± 12.0^a^	59.3	0.766^b^	61.9	0.940^b^	63.8	0.286^b^
	> 60		53.3		58.2		79.1	
Sex	Female	17 (13.7)	61.8	0.830^b^	61.8	0.921^b^	67.7	0.926^b^
	Male	107 (86.3)	57.5		61.4		70.6	
Subsite	Base of tongue	94 (75.8)	57.6	0.807^b^	63.4	0.829^b^	68.7	0.756^b^
	Soft palate	22 (17.7)	36.4		40.4		80.8	
	Tonsil	6 (4.8)	66.7		66.7		66.7	
	Pharyngeal walls	2 (1.6)	100		100		100	
T stage	T1	15 (12.1)	73.3	0.421^b^	86.7	0.024^b^	86.7	0.018^b^
	T2	36 (29.0)	55.2		62.4		72.8	
	T3	46 (37.1)	52.8		50.1		55.7	
	T4	27 (21.8)	63.3		76.7		85.2	
N stage	N0	76 (61.3)	61.9	0.003^b^	62.8	0.712^b^	70.8	0.749^b^
	N1	28 (22.3)	55.9		49.0		54.8	
	N2	16 (12.9)	60.0		71.4		71.4	
	N3	4 (3.2)	25.0		50.0		50.0	
SND	I-III	62 (50.0)	55.0	0.914^b^	58.9	0.778^b^	74.0	0.290^b^
	I-IV	62 (50.0)	60.1		61.5		64.8	
DOI, mm	≤ 5	21 (16.9)	63.0	0.546^b^	62.1	0.864^b^	62.1	0.666^b^
	5 < DOI ≤ 10	30 (24.2)	40.6		51.7		64.6	
	> 10	73 (58.9)	62.3		65.8		72.1	
Histologic grade	Well	62 (50.0)	56.8	0.871^b^	62.0	0.531^b^	69.2	0.768^b^
	Moderate	54 (43.5)	50.0		68.2		70.2	
	Poor	8 (6.5)	75.0		75.0		75.0	
LVI	Yes	14 (11.3)	50.0	0.786^b^	65.0	0.600^b^	75.0	0.673^b^
	No	110 (88.7)	59.5		45.2		68.0	
PNI	Yes	20 (16.1)	70.0	0.646^b^	80.0	0.524^b^	80.0	0.783^b^
	No	104 (83.9)	56.2		59.0		69.5	
Radiotherapy	Yes	61 (49.2)	63.9	0.421^b^	70.8	0.235^b^	75.5	0.415^b^
	No	63 (50.8)	53.8		57.6		66.4	
Chemoradiotherapy	Yes	20 (16.1)	44.0	0.071^b^	26.8	0.019^b^	26.8	0.005^b^
	No	104 (83.8)	61.4		70.8		79.9	

*Abbreviations*: *DFS* Disease-free survival, *OS* Overall survival, *DSS* Disease-specific survival, *P p* value, *SND* Selective neck dissection, *DOI* Depth of invasion, *LVI* Lymphovascular invasion, *PNI* perineural invasion

^a^Mean ± SD

^b^A log-rank test

### Distribution of lymph node metastasis

**Fig. 2:**
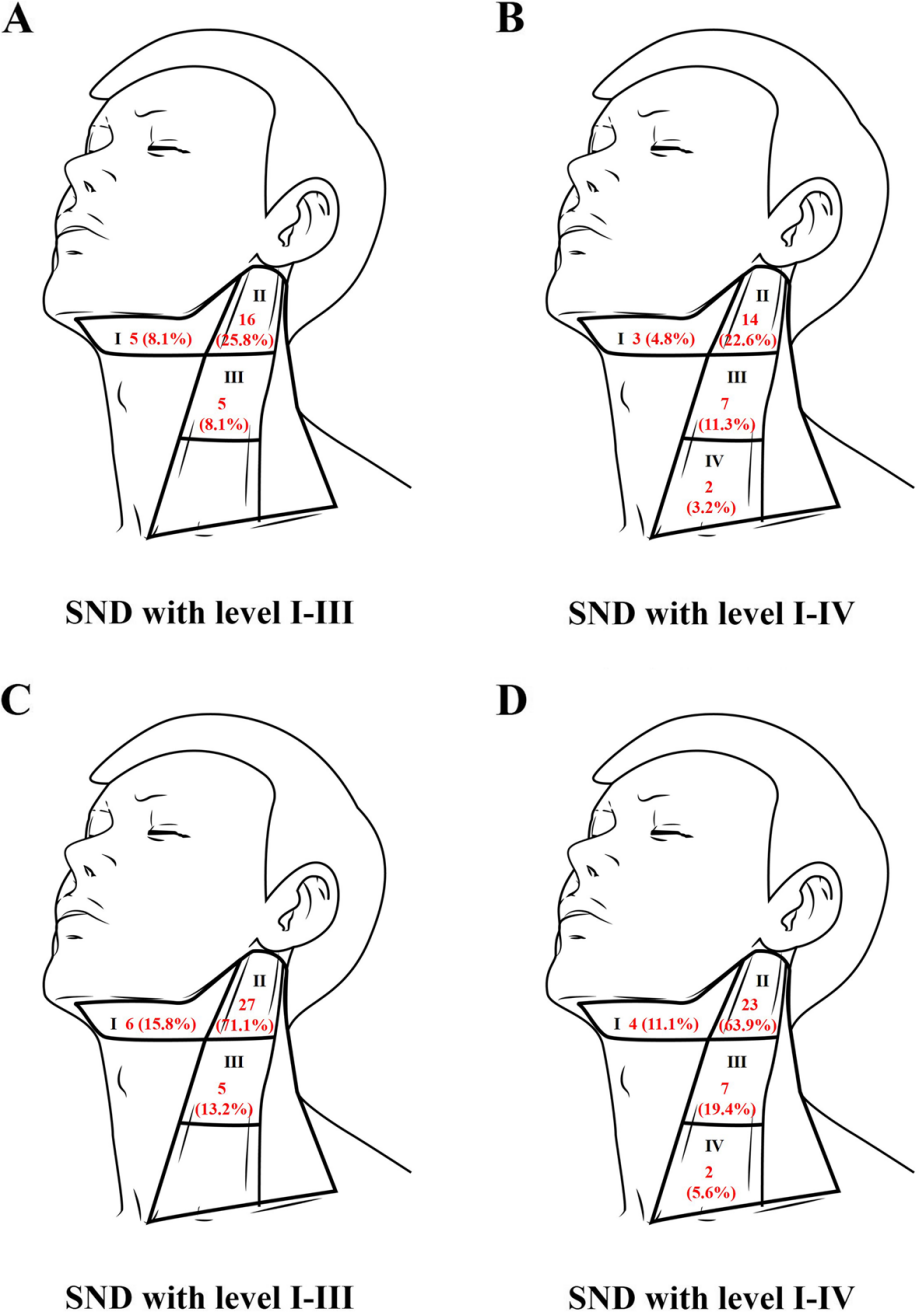
Lymph nodes metastasis in each level of ipsilateral neck (**A**) Number of patients who occurred lymph node metastasis in each level from 62 SNDs with level I-III; (**B**) Number of patients who occurred lymph node metastasis in each level from 62 SNDs with level I-IV. **C** Number of metastatic lymph nodes in each level from 62 SNDs with level I-III; (**D**) Number of metastatic lymph nodes in each level from 62 SNDs with level I-IV. Drawings in Fig. 2 were depicted by our own

For the entire cohort, 112 patients underwent unilateral neck dissection, and 12 patients (4 patients in Group A and 8 patients in Group B) underwent bilateral neck dissection (all contralateral neck dissections were level I-III). Forty-eight patients (38.7%) had occult lymph node metastasis, among which none was found in the contralateral neck (0/12). N1 accounted for 28 cases (22.6%) and N2 and N3 for 16 (12.9%) and 4 (3.2%, all cases of N3 were N3b) (Table 1). In Group A, occult lymph node metastasis rates of the ipsilateral neck at levels I, II and III were 8.1% (n = 5), 25.8% (n = 16) and 8.1% (n = 5), respectively (Fig. 2A). In Group B, metastasis rates of the ipsilateral neck in level I-IV were 4.8% (n = 3), 22.6% (n = 14), 11.3% (n = 7) and 3.2% (n = 2) (Fig. 2B). Two cases of level IV metastasis originated from the base of tongue (pT4aN2bM0, DOI > 10 mm, grade II) and the lateral pharyngeal wall (pT3N2bM0, DOI > 10 mm, grade II). In level IV, they both had only one intranodal metastatic lymph node. The case located in base of tongue had invaded midline and had one metastatic lymph node in level II (ENE-). Another one had no midline involvement and both had one metastatic lymph node in level II (ENE-) and level III (ENE-). Regarding the distributed numbers of metastatic lymph nodes of the ipsilateral neck, there were 6 (15.8%), 27 (71.1%) and 5 (15.8%) lymph nodes at levels I, II and III in Group A (Fig. 2C) and 4 (11.1%), 23 (63.9%), 7 (19.4%) and 2 (5.6%) lymph nodes in Group B (Fig. 2D).

### Group comparison of regional control and survival analysis

The median follow-up time was 46 months (range, 22–71). At the last follow-up, only 1 patient (1/62, 0.8%) had ipsilateral neck recurrence, which occurred at level II (Group A); it was confirmed as lymph node metastasis by pathology. Group B had no recurrence in the ipsilateral neck (0/62, 0%). No recurrence in the contralateral neck was found, though 2 patients in Group B experienced contralateral metastasis at level II after ipsilateral neck dissection.

**Fig. 3:**
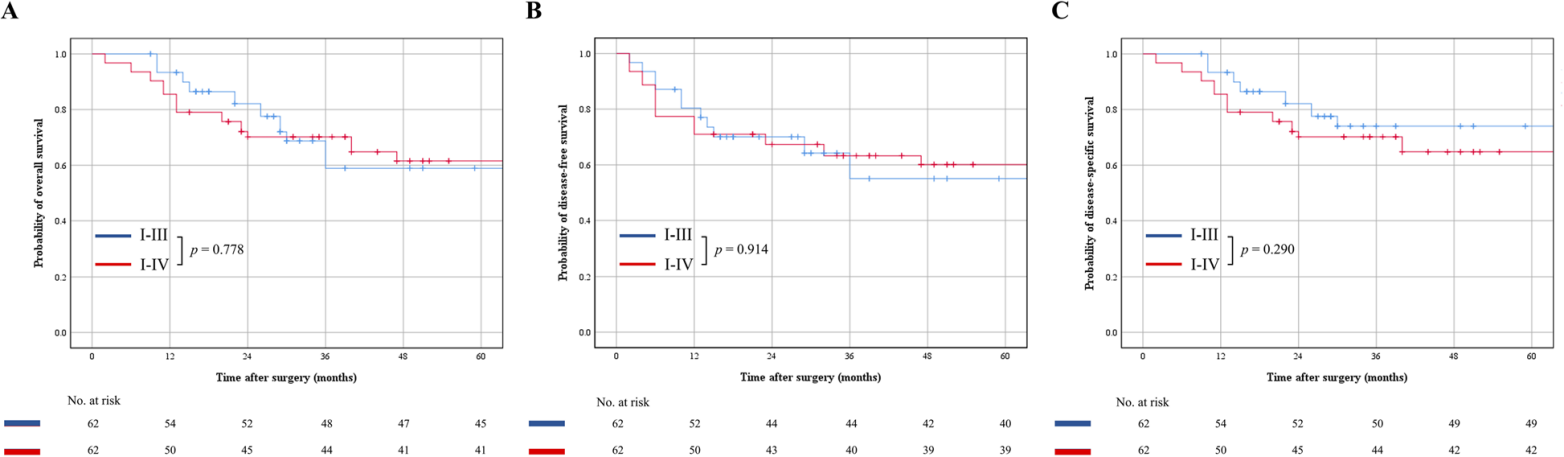
Overall comparison of (**A**) disease-free survival, (**B**) overall survival and (**C**) disease-specific survival between the group that performed with selective neck dissection of I-III and I-IV. *p* value was estimated by a log-rank test

For the entire cohort, the 5-year DFS, OS and DSS rates were 58.5%, 61.1% and 68.4%, respectively (Table 3). The 5-year DFS rate was 55.0% in Group A and 60.1% in Group B (*p* = 0.914) (Table 3 & Fig. 3A). The five-year OS rate of Group A was 58.9%, similar to that of Group B (61.5%, *p* = 0.778) (Table 3 & Fig. 3B). The five-year DSS rate was 74.0% in Group A and 64.8% in Group B (*p* = 0.290) (Table 3 & Fig. 3C).

### Univariate analysis

In univariate survival analysis, there was no significant difference between Group A and Group B (SND) with regard to 5-year DFS, OS and DSS (Table 3 & Fig. 3). N stage was the only significant factor associated with 5-year DFS (*p* = 0.003), and patients with N0-stage disease exhibited the highest 5-year DFS rate, at 61.9% (Table 3). Five-year OS correlated with T stage (*p* = 0.024) and adjuvant chemoradiotherapy (*p* = 0.019) (Table 3). Patients with T1 stage disease had the highest 5-year OS rate of 86.7%, whereas these patients did not benefit from adjuvant chemoradiotherapy (26.8%) (Table 3). The five-year DSS rate was related to T stage (*p* = 0.018) and chemoradiotherapy (*p* = 0.005) (Table 3). Similar to OS, T1 stage had the highest 5-year DSS rate, at 86.7%, and patients who underwent adjuvant chemoradiotherapy had a 5-year DSS rate of only 26.8% (Table 3). Adjuvant radiotherapy led to distinct improvements in DFS (63.9% vs. 53.8%, *p* = 0.421), OS (70.8% vs. 57.6%, *p* = 0.235) and DSS (75.5% vs. 66.4%, *p* = 0.415), none of which showed statistical significance (Table 3).

### Multivariate survival analysis

SND and other covariates, which were screened from univariate analysis, were included in Cox proportional hazard models for multivariate analysis of 5-year DFS, OS and DSS. The results showed that SND was not an independent prognosticator for 5-year DFS (HR 1.02, *p* = 0.949), OS (HR 0.88, *p* = 0.731) or DSS (HR 1.42, *p* = 0.354) (Table 4).

**Table 4:** Multivariate survival analysis

DFS	B	P	HR	95% CI HR	
				Inf	Sup
**SND (I-IV)**	0.019	0.949	1.019	0.564	1.841
**N stage**		0.185			
N1	0.200	0.583	1.221	0.598	2.495
N2	0.302	0.510	1.353	0.551	3.332
N3	1.357	0.030	3.883	1.140	13.224
**Chemoradiotherapy**	0.727	0.041	2.068	1.029	4.155
**OS**	**B**	**P**	**HR**	**95% CI HR**	
				Inf	Sup
**SND (I-IV)**	-0.131	0.731	0.878	0.418	1.845
**T stage**		0.115			
T2	0.061	0.940	1.063	0.214	5.283
T3	0.957	0.223	2.605	0.559	12.142
T4	0.174	0.843	1.190	0.214	6.622
**Radiotherapy**	-0.785	0.055	0.456	0.205	1.015
**Chemoradiotherapy**	0.880	0.042	2.411	1.031	5.636
**DSS**	**B**	**P**	**HR**	**95% CI HR**	
				Inf	Sup
**SND (I-IV)**	0.353	0.354	1.424	0.674	3.007
**T stage**		0.278			
T1	0.172	0.833	1.187	0.242	5.831
T2	0.847	0.280	2.332	0.502	10.823
T3	0.075	0.930	1.078	0.200	5.819
**Chemoradiotherapy**	0.700	0.079	2.013	0.922	4.395

Cox proportional hazard model was used for multivariate survival analysis

*Abbreviations*: *DFS* Disease-free survival, *SND* Selective neck dissection, *OS* Overall survival, *DSS* Disease-specific survival, *B* β coefficient, *P p* value, *HR* Hazard ratio, *95% CI* 95% Confidence Interval

N3 stage (HR 3.89, *p* = 0.030) and chemoradiotherapy (HR 2.07, *p* = 0.041) were both related to worse 5-year DFS (Table 4). Chemoradiotherapy, an independent prognosticator (HR 2.41, *p* = 0.731) for 5-year OS, correlated with a worse survival outcome. Conversely, T stage (*p* = 0.155) and radiotherapy (HR 0.46, *p* = 0.055) did not correlate with OS (Table 4). In multivariate analysis of 5-year DSS, no independent prognosticator was found (Table 4).

## Discussion

For cN + OPSCC, SND, including level IV, has been widely accepted. A multi-institutional retrospective review validated that an SND approach incorporating level II-IV with postoperative adjuvant therapy provides a long-term regional control rate of 97.4% (4/151) and a 5-year OS rate of 88% for cN1-cN3 HPV-positive OPSCC [9]. Mendez et al. reported that patients with HPV-positive OPSCC treated with transoral robotic surgery and unilateral neck dissection of II-IV, followed by the indicated adjuvant therapy, can have a 2-year DFS rate of 95% and a 2-year OS rate of 100% [20]. According to the American National Cancer Database of 2358 HPV-positive OPSCC cases, 11.9% of patients (230/1935) who receive therapeutic neck dissection experience lymph node metastasis at ipsilateral level IV. For patients who undergo END, only 4.0% (17/423) have occult lymph node metastasis at level IV. Hence, the authors considered that therapeutic neck dissection should encompass at least level II, III, and IV and that END encompassing level II-III is sufficient for adequate pathologic staging and treatment of HPV-positive OPSCC [10].

Nonetheless, research on level IV neck dissection in cN0 OPSCC remains controversial. Evidence to date largely relates to the distribution of cervical lymph node metastasis based on observational studies. Moreover, the distribution of level IV lymph node metastasis in cN0 OPSCC remains debatable due to evidence arising from limited sample sizes. Ballantyne et al. reported relative percentages of metastatic nodes at level IV in the cN0 neck from the base of the tongue and pharyngeal walls of 33% and 40%, respectively [21]. En Chang Choi et al. found an incidence of lymph node metastasis at level IV in cN0 OPSCC patients of 3.0% (1/33) in the ipsilateral neck and 3.8% (2/52) in the contralateral neck. All 3 cases were seen in combination with metastasis at level III [2]. Kowalski et al. retrospectively reviewed 22 cN0 OPSCC cases treated with radical neck dissection, with no metastasis at level IV. Furthermore, the rate of metastasis at level IV was only 3.4% (2/59) for cN + OPSCC [7]. Califano et al. also found that the risk of occult lymph node metastasis in level IV tended to be < 5% when level III was radiologically negative. Therefore, exclusion of level IV is an alternative choice for sparing irradiated volume when level III is pathologically negative in both cN0 and cN + HPV-positive OPSCC [22]. In a prospective analysis of 24 cN0 OPSCC patients, 8 experienced lymph node metastasis in the ipsilateral neck, none of which occurred at levels III and IV [23]. In general, despite being in dispute, there largely appears to be a low incidence of metastasis at level IV for cN0 OPSCC in previous reports. According to our data, patients with cN0 HPV-negative OPSCC have a lower metastasis rate of 3.2% (2/62) at level IV when undergoing I-IV neck dissection. The distribution was close to that of Choi et al.

In addition, the distribution of lymph nodes in the neck is not evidence of a high level. The rate of regional control and survival outcomes, especially for level IV patients, from cohort studies or randomized controlled studies are convincing, though such statistics are seldom used. Ryan et al. reported 21 ENDs of p16 + OPSCC containing occult lymph node metastasis in 5 cases (23.8%), which all presented at level II. Following the indicated adjuvant treatment, SND with IIA-IIB and III was associated with a low nodal recurrence rate [24]. Our cohort study authenticated that when compared with SND of I-IV, SND of level I-III does not increase the probability of regional recurrence at level IV and that 5-year OS, DSS and DFS rates do not decline.

Excluding level IV from neck dissection would avoid some surgical complications. Chylous leakage is infrequent but potentially lethal, leading to skin necrosis and exposure of the carotid artery. The morbidity of this complication has been reported to range from 1%-5.8% [25]. Our study showed that including neck dissection of level IV would slightly increase the incidence of chylous leakage and hematoma. However, there were no differences between the groups in which patients had phrenic nerve paralysis and wound infection.

Thus, excluding level IV might be an alternative algorithm of SND for cN0 HPV-negative OPSCC. Nevertheless, should it be safety omitted for SND of all kinds of cN0 HPV- OPSCCs? En Chang Choi et al. considered that the incidence of metastasis at level IV for cN + OPSCCs was significantly higher for tumor at base of tongue (6/7, 85.7%) compared with tonsillar cancer (20/59, 33.9%) [2]. Kowalski et al. found metastasis rate for cN0 neck of ipsilateral level IV was higher in vallecula (3/7, 42.9%) than tonsil (6/55, 10.1%), base of tongue (0/16, 0%) and soft palate (0/3, 0%) [7]. Since base of tongue and vallecula are located adjacent to each other, and 1 of our 2 cases occurring level IV metastasis was located in base of tongue, this might be a high-risk area for level IV metastasis. Hyang et al. deemed that occult lymph node metastasis at level IV was mostly occurred at ipsilateral neck with primary of T3-T4 stage. Therefore, neck dissection of level IV was unnecessary for patient with primary below T3 stage and contralateral neck, which is in accordance with our outcome that the 2 cases were T3 and T4 stage [25]. There were also some other risk factors that a univariate logistic regression revealed that level III involvement predicted the presence of disease in level IV [22]. While skip metastasis should also be precaution. Further study is still needed for more precise risk factors when treating cN0 OPSCC.

An evidence-based guideline from the American Society for Radiation Oncology (ASTRO) states that patients with OPSCC should not routinely receive concurrent systemic therapy with postoperative radiotherapy but that it should be given with some specific indications [26]. Duvvuri et al. reported that adjuvant radiotherapy or chemoradiotherapy did not provide a survival benefit for early-stage OPSCC with low- or intermediate-risk pathologic features [27]. Concurrent chemotherapy is associated with improved OS for patients with lymph node-positive OPSCC but not for lymph node-negative OPSCC and stage I HPV-positive OPSCC undergoing definitive radiotherapy [28]. Liu et al. demonstrated that platinum-based chemotherapy to conventional radiotherapy reduces the risk of distal metastasis for 15% of HPV-positive OPSCCs with higher risk (T4 and/or N3) but not for HPV-negative patients [29]. Our data showed that chemoradiotherapy correlates with worse DFS and OS. In fact, few patients received chemoradiotherapy (20 of 124), and stratification analysis for chemoradiotherapy was limited. Hence, indications for adjuvant chemoradiotherapy require further study with large and stratified samples.

Our study also has some limitations. First, the study was primarily related to a retrospective design and nonrandom treatment assignment. The choice of SND was subjective since it was based on the experience and preference of each surgeon, which may lead to bias. Although a patient might not have a survival benefit when including neck dissection of level IV, excluding this level should also be prudent, and a large sample prospective study is needed because patients with lymph node metastasis or recurrence at level IV have a worse survival rate than patients with other neck levels [2]. Second, the OPSCC cases mainly were in the base of the tongue, and subsites of the soft palate, tonsil and pharyngeal walls were lacking. In addition, few cases were enrolled in this cohort because HPV status was not assessed before 2015 in our hospital.

## Conclusions

In conclusion, the results provide evidence that SND, including level IV SND, does not contribute to improved survival outcomes in cN0 HPV-negative OPSCC. However, a large-sample prospective stratified study is needed to explore the survival benefit of adjuvant chemoradiotherapy for HPV-negative OPSCC.

## Data Availability

The datasets used and/or analysed during the current study are available from the corresponding author on reasonable request.

## Abbreviations

OPSCC: Oropharyngeal squamous cell carcinoma
HPV: Human papillomavirus
SND: Selective dissection
DFS: Disease-free survival
OS: Overall survival
DSS: Disease-specific survival
END: Elective neck dissection
IHC: Immunochemistry
ISH: In situ hybridization
LVI: Lymphovascular invasion
PNI: Perineural invasion
DOI: Depth of invasion
ASTRO: American Society for Radiation Oncology
